# Antimicrobial activity of *Salvia spinosa* against *Enterococcus faecalis* causing endodontic infections: an in-vitro, ex-vivo, and in-silico study

**DOI:** 10.1186/s12906-025-04983-y

**Published:** 2025-07-09

**Authors:** Wedad M. Nageeb, Sherouk Hussein Adam, Nihal Ali, Marwa Sharaan

**Affiliations:** 1 Department of Medical Microbiology and Immunology, Faculty of Medicine, Suez Canal University, Ismailia, Egypt; 2 Department of Endodontics, Faculty of Dentistry, Suez Canal University, Ismailia, Egypt; 3 Directorate of Health, Suez, Egypt

**Keywords:** *Enterococcus faecalis*, *Salvia spinosa*, Antibacterial, Calcium hydroxide, Endodontic medications, Root canal medications, Herbal alternatives

## Abstract

**Background:**

*Enterococcus faecalis (E. faecalis)* often persists in root canal systems and it is rather unsusceptible to many root canal medications including the standard Calcium Hydroxide treatment which often causes therapeutic failure. Natural herbal alternatives are preferred as new intracanal medications since they are less-toxic, renewable, and are cost-effective. The aim of this work was to assess the effectiveness of *Salvia spinosa* and its combination with Calcium Hydroxide as intracanal medications for eliminating *Enterococcus faecalis* in an ex-vivo root canal infection model compared to standard Calcium Hydroxide treatment.

**Methods:**

*Salvia spinosa* methanolic extract was tested using the standard disc diffusion method against *Enterococcus faecalis* ATCC 19433 and compared to Ca(OH)_2_ and their combination. Forty-four extracted teeth were divided into 3 experimental groups and a control group. An ex-vivo model of endodontic teeth infection was established to test the effect of *S. spinosa* and its combination with Ca(OH)_2_. against *E. faecalis.* Further in-silico testing of the interaction of 19 *S. spinosa* active constituent with 9 important *E. faecalis* surface adhesins and virulence proteins was performed using Autodock tools.

**Results:**

*S. spinosa* extract and its combination with Ca(OH)_2_ possess similar antibacterial effects which were significantly higher than conventional Ca(OH)_2_. Flavone, Flavonone, Spathulenol, and Caryophyllene oxide are the best of the tested binding active substances of *S. spinosa* to most of the Enterococcal surface proteins studied. Both Enterococcal surface protein (6ORI) and the collagen adhesion protein (2Z1P) could act as important *E. faecalis* drug targets.

**Conclusion:**

*S. spinosa* shows promising antimicrobial activity against *E. faecalis* and thus offers a promising natural alternative approach to treat root canal endodontic infections for further detailed testing and investigation.

**Supplementary Information:**

The online version contains supplementary material available at 10.1186/s12906-025-04983-y.

## Introduction

*Enterococcus faecalis* has been identified as a primary contributing factor to the failure of endodontic treatment. It is recognized for its high resistance to various disinfectants and antimicrobials, leading to the persistence of extra-radicular and intra-radicular infections [1, 2]. It is principally identified in persistent endodontic infections and also in asymptomatic infections with prevalence ranging between 24 and 77% [3].

The persistence of *E. faecalis* in root canal infections is ascribed to various mechanisms, including its array of virulence factors and surface adhesion proteins, as well as its competition with other bacterial species, which modifies host responses and leads to infections that are challenging to eliminate [3, 4]. *E. faecalis* is known for its resistance to antibiotics, detergents, heavy metals, and bile salts. It can also resist the effect of desiccation, ethanol, and azide. It can survive in extremely harsh environmental conditions including increased pH, low nutrient levels, and high salt concentrations [5]. About 63% of root canal treatment failures experienced reinfection commonly caused by *E. faecalis* [6]. *Enterococcus faecalis* can persist in root canal systems, leading to recurrent infections due to its capacity to tolerate nutritional deprivation, its infiltration of dental tubules, its adhesion to dentin, and its ability to inhibit lymphocyte activity, so altering the host response [7].

It has been suggested that Calcium Hydroxide (Ca(OH)_2_), the standard intracanal medication, is ineffective in the elimination of *E. faecalis* [8]. *E. faecalis* can survive intracanal treatment with Ca(OH)_2_ due to its ability to maintain PH haemeostasis through pumping protons and maintaining a lower internal PH, thus avoiding the alkaline environment created by Ca(OH)_2_ [3]. In addition to its low effectiveness against *E. faecalis*, Ca(OH)_2_ has many disadvantages as an intracanal medication which warrants a search for a natural less- toxic alternatives effective in the treatment of *E. faecalis.* This alternative should have lower potential of developing resistance compared to other antimicrobial drugs.

*Salvia* genus contains widely distributed species which have been used in folk medicine as well as pharmaceutical and food industries [9]. The genus *Salvia* is the largest genus from the Lamiaceae family showing worldwide distribution of nearly 1000 species. *Salvia spinosa* is a polymorphic taxon with high morphological variability [10]. *Salvia* has been used in traditional medicine showing a wide range of anti-inflammatory, antioxidant, anti-diabetic, anti-tumor, antiangiogenic, hepatoprotective, neuroprotective, sedative, anxiolytic, anti-neuropathic, and antibacterial activity [11]. *Salvia spinosa* is commonly utilized for its medical properties, including treatment for stomach disturbances, anti-inflammatory gargles, cough suppression, antiseptic applications, anti-rheumatic effects, astringency, and relief from hemorrhoidal pain. It is also used as a carminative and hypotensive agent [12].

Plants provide a precious supply of secondary metabolites offering a wide range of bioactivities in different biologic systems. This genus is a valuable resource of bioactive compounds including phenolics, flavonoids, and terpenoids [13]. These substances are thought to be primarily responsible for *Salvia* medicinal activities. About 19 active compounds have been identified from *Salvia spinosa* including; Thymol, Terpinene, Methyl chavicol, Caryophyllene, Spathulenol, linalool, trans-caryophylle, α-terpinolene, Isopentyl isovalerate, Isopentyl 2-methyl butanoate, β-ocimene, β-patchoulene, β-bourbonene, Germacrene D, α-Gurjunene, β-Gurjunene and 1,8- cineol as the major active constituents from *S. spinosa* [9, 13–16]. Flavones and flavanones have been identified as the major flavonoid classes from *S. spinosa* [17]. Caryophyllene oxide, Spathulenol, and Linalool have been identified to occur at high concentrations from *S. spinosa* extract in other studies [13].

Medicinial use of plant extract is gaining increased interest as a new alternative to tackle resistant microbes. Antibacterial activity of *Salvia spinosa* has been reported against *Pseudomonas aeruginosa*, *Staphylococcus aureus*, and *Bacillus subtilis* [18]. Another study has also demonstrated a promising antibacterial efficacy of *Salvia spinosa* as a natural antibacterial agent against *S. aureus*, *S. pneumoniae*, *E. coli*, and *B. cereus* [19]. On the other hand, some other studies demonstrated the absence of antibacterial effect of *Salvia spinosa* against 5 tested isolates including *Ps. aeruginosa*, *E. coli*, *Klebsiella*, *Enterobacter*, *S. aureus* [20]. Recent studies have demonstrated the promising in-vitro antibacterial effect of *S. spinosa* against *S. aureus* and *E. coli* [21].

Finding an effective method to eliminate *E. faecalis* from root canal systems is currently challenging especially with the reported resistance of the organism to antibiotics which are commonly used in dental treatment including erythromycin and tetracycline [22]. Using herbal extracts offers a viable approach to tackle such resistant organisms, especially with infections requiring local applications of the herbal medications which provide reduced exposure to any possible side effects. No studies are available about the effect of *S. spinosa* against *E. faecalis*.

In addition to using natural alternatives in endodontic treatments, combination therapy with herbal medications also provides a promising approach to enhance therapeutic effectiveness while reducing toxicity and undesirable effects at the same time.

The aim of this study was to investigate the antibacterial effect of *Salvia spinosa* and its combination with conventional Calcium Hydroxide treatment against *Enterococcus faecalis* causing root canal infections and to test for the potential of in-silico interaction of its active components to target *Enterococcus faecalis* important surface adhesion proteins and virulence factors. In this work, we test the antibacterial effect of *S. spinosa* against *E. faecalis* in-vitro and also using an ex-vivo model and we compare its effect to the effect of Ca(OH)_2_ and to the effect of *S. spinosa* and Ca(OH)_2_ combination. We also perform an in-silico study of 19 active substances reported from *S. spinosa* against 9 *E. faecalis* important surface proteins. To the best of our knowledge, no previous studies have investigated the effect of *Salvia spinosa* extract and its combination with Calcium Hydroxide against *Enetrococcus faecalis* and its use as a potential intracanal medication.

## Materials and methods

### Ethical approval

Performance of the study was approved by the Institutional Review Board and the research and ethics committee of the Faculty of Dentistry, Suez Canal University, for performing the experiments, (Ethical Committee Serial Number 424/2021). All methods and experiments were performed according to relevant guidelines and regulations. All experimental protocols were approved by the Institutional Review Board of the Faculty of Dentistry, Suez Canal University. The study was waived from ethical approval since it was conducted on unidentified random extracted premolars not linked to specific patients’ identities which have been already extracted in the clinic due to other treatment reasons such as for periodontal or orthodontic purposes. Informed consent was obtained from all subjects.

### Plants materials

The aerial parts of the plant samples of *Salvia spinosa* L. were collected during March - April 2023 from Saint Catherine, South Sinai, Egypt (Coordinates: 28.5619° N 33.9493° E, Elevation: 1,565 m (5,135 ft) above the sea level) with prior permission from the landowner. The climate in this area is characterized by arid and dry desert weather. According to the Köppen-Geiger classification, the prevailing climate in this region is categorized as BWk. Formal identification of the collected plant material has been carried out by Prof. Dr. Ashraf Mohamed Mohamed Khalil, Prof. of Medicinal and Aromatic plants, Horiculture Research Institute, Agriculture Research Centre, Giza, Egypt. The material of the plant used is deposited and available from the National Network of Egyptian Herbaria reference 511(1771).

### Preparation of extracts

Methanol (MeOH) extracts of the powdered aerial parts of *S. spinosa* were prepared by the maceration method. *Salvia* plant aerial parts were finely ground using an electric grinder. A 750 gm of ground powder was soaked in absolute methanol to extract existing metabolites. This cold maceration process was repeated three times (3 × 3 L) at ambient temperature with a sonicator aid to ensure complete extraction. The combined methanolic solutions were concentrated under vacuum to afford 60 g of *Salvia spinosa* extract [23]. The extract was stored at 4 ℃ till used.

### In-vitro antimicrobial susceptibility study of the effect of *S. Spinosa* extract against *E. faecalis*

The disc diffusion method, Kirby-Bauer test, was used as a standardized technique to evaluate the antimicrobial susceptibility of the reference *Enterococcus faecalis* strain (ATCC 19433) against the tested drugs. A bacterial suspension was prepared from a pure culture, adjusting its turbidity to match a 0.5 McFarland standard and then evenly spread in lawn culture using a sterile swab over the surface of Muller-Hinton agar. Sterile filter paper discs impregnated with the test material (*S. spinosa* 25% extract, *S. spinosa* 12.5% extract, Calcium Hydroxide 4%, Calcium Hydroxide 2%, Combination of *S. spinosa* 25% extract and Calcium Hydroxide 4%, and Combination of *S. spinosa* 12.5% extract and Calcium Hydroxide 2%). The discs impregnated with different material were placed on the surface of the inoculated agar plates using a sterile forceps. The discs were evenly spaced to prevent overlapping zones of inhibition. The plates were then incubated at 35–37 ºC for 18–20 h. Diameters of inhibition zones were then recorded to determine susceptibility against the tested compounds. The test was performed in triplicates.

### Ex-vivo root Canal system endodontic model study of the effect of *S. Spinosa* extract against *E. faecalis*

The study was performed on unidentified 44 extracted premolar teeth which were divided randomly into four groups according to type of medication tested. All premolars were extracted, cleaned from outside to remove any soft or hard tissue debris by scaler, then washed with water, and stored in sterile saline solution till used in the study.

The unidentified 44 extracted premolar teeth were divided randomly into four groups according to type of medication as follows:

1. Twelve roots were filled with 25% Salvia spinosa extract

Group 2 Twelve roots were filled with calcium hydroxide 4% solution.

Group 3 Twelve roots were filled with a mixture of calcium hydroxide 2% and 12.5% Salvia spinosa extract.

Group 4 Eight roots were filled with standard calcium hydroxide paste (Control Group).

### Teeth Preparation

De-coronation of the selected teeth at cementoenamel junction was made using a diamond bur mounted on Low-speed micro motor under water coolant leaving standardized roots length of approximately 12 mm. All root canal samples were prepared by ProTaper NiTi rotary system according to the manufacturer’s instructions. The working length was visually determined by Introducing a size #10 K-file into the root canal until its tip was observed at the apical foramen; and then subtracting 1 mm from that value and the root canal diameter was standardized by selecting all the canals with initial apical file# 25 K-file [24]. Completion of roots preparation was done by using Protaper files until size F4 using an Endo motor. In between each step the canals were thoroughly irrigated with 2 ml, 2.5% NaOCl, followed by 2 ml of saline to remove all dentine debris. All root apices were covered with resin composite. Then all roots were sterilized in autoclave (at 121 °C, for 20 min). Sterility of tested teeth was confirmed prior to conducting the study.

### Inoculation of root canals with *E. faecalis*

Under aseptic conditions, 20–40 µl of 0.5 McFarland *E. faecalis* bacterial suspension was injected into each root canal to the entire root canal length, until the entire canal space was filled with fluid. After injection, each specimen was completely submerged in pre-sterilized Brain Heart Infusion (BHI) broth in a microtube which was then incubated at 37 ºC for 21 days. Every 3 days, 0.3 ml sample was taken out and 0.5 ml of fresh BHI broth was added to ensure bacterial viability [25].

### Base line root Canal sampling

At the end of the incubation period, the roots were removed from the tubes using sterile forceps and washed three times with 5 ml of sterile saline both inside and outside the root canal. After that, samples were transferred into sterile microplate. The first bacteriological (Baseline) sampling was taken before intra canal medication placement using sterile paper points. Two sequential pre-sterilized paper points were placed inside each root canal, each for 2 min. After sampling, each paper point was transferred to a microtube containing 1 ml of sterile (BHI) broth. The tubes were then shaken on a vortex for 30 s. A 0.1 ml aliquot of the microbial suspension was spread and plated then serially diluted and incubated on TSA agar plates for 48 h at 37 ºC. After confirming the purity of the positive cultures using Gram staining and colony morphology, the number of colony forming units (CFUs) of each specimen was determined from the average of the three serial dilutions for each specimen [26].

### Intracanal treatment of the root canals

The forty-four roots were randomly divided into four groups and the medications were applied in root canals under sterile conditions with the aid of an automatic micropipette then a temporary filling material was placed at the opening of each canal as a seal. Each experimental group was then incubated for 7 days or 14 days at 37 ºC.

### Post-medication bacterial sampling from root canals

Samples from each experimental group were taken at the two incubation time points. The intracanal treatment was removed using a sterile K#25 file and the canal was irrigated with 5 ml of sterile saline using a disposable syringe. The content of the root canal was absorbed into a sterile paper point. Two consequent paper points were used, and each paper tip was maintained in position inside the canal at the established working length for 1 min and then transferred into a microtube containing 1 ml of Brain Heart Infusion (BHI) broth. Each sample was serially diluted, and a volume of 0.1 ml was spread from each dilution on TSA agar. From each sample, the bacterial counts of serial dilutions were performed on TSA plates after a two-day incubation at 37 °C.

### In-silico study of the interaction between active constituent of *S. Spinosa* and *E. faecalis* surface adhesion proteins

In-silico testing of interaction of *S. spinosa* active constituent was tested with important *E. faecalis* protein macromolecules as previously described [27]. The 3D structure of 19 different ligands that may act as active drug components was downloaded from PubChem database at https://pubchem.ncbi.nlm.nih.gov/. Target compounds tested included Linalool (PubChem CID: 6549), beta-Caryophyllene oxide (PubChem CID: 1742210), Spathulenol (PubChem CID: 92231), Thymol (PubChem CID: 6989), alpha-Gurjunene (PubChem CID: 15560276), Isopentyl isovalerate (PubChem CID: 12613), Methyl chavicol (Estragole) (PubChem CID: 8815), Flavones (PubChem CID: 10680), flavanone (PubChem CID: 10251), 1,8-Cineol(Eucalyptol) (PubChem CID: 2758), o-Cymene (PubChem CID: 10703), Limonene (PubChem CID: 22311), α-terpinolene (PubChem CID: 11463), γ-Terpinene (PubChem CID: 7461), β-ocimene (PubChem CID: 18756), β-patchoulene (PubChem CID: 101731), Sabinene (PubChem CID: 18818), alpha-PINENE (PubChem CID: 6654), and beta-Pinene (PubChem CID: 14896).

Three target surface proteins macromolecules adhesins from *Enterococcus faecalis* were downloaded from PDB (Protein Data bank) database including Enterococcal surface protein (6ORI), Adhesin domain of PrgB (6EVU), and collagen adhesion protein (2Z1P). Six other Enterococcal proteins predicted structures were downloaded from AlphaFold [28] including Enterococcal surface protein *esp* (F6MHT4), Peptide pheromone-binding protein TraC (A0A6I4Y5I4), *Enterococcus faecalis* protein Asa1 (M1TU09), Endocarditis and biofilm-associated pilus major subunit EbpC (A0A7H0FRK3), collagen adhesin (Ace) (E1ART9), and Aggregation substance PrgB/Glucan-binding protein (M1U904). Target proteins were prepared by removal of water, adding kollman charge and polar hydrogrns and removal of ions, cofactors, ligands, or other molecular structures using MGLtools version 1.5.7. and Autodock 4 and [29, 30].

Autodock was used to perform molecular docking for all *S. spinosa* tested ligands with 9 Enterococcal target proteins. Analysis and visualization of output results was performed to find which interaction had the highest RMSD (Root Mean Square Deviation), higher binding affinity and conditions of hydrogen bonds as well as other types of bonding was performed using Proteins*Plus* available at https://proteins.plus [31, 32], protein ligand profiler [33, 34], and LigPlot^+^ v.2.2 [35].

### Statistical analysis

Data was entered into Microsoft Excel sheets for analysis. Mean and Standard Deviation were used to describe normally distributed data. A two-way between-groups analysis of variance (ANOVA) was conducted to explore the impact of the type of intracanal medication used and time interval of medication application on counts of *Enterococcus faecalis* as measured by percent reduction in live colony counts. Post-hoc comparisons were performed using the Dunnett test and also the Tukey Post hoc HSD comparison. Statistical analysis was performed using SPSS Statistical package for analysis (SPSS^®^ sofware, IBM).

## Results

**Fig. 1:**
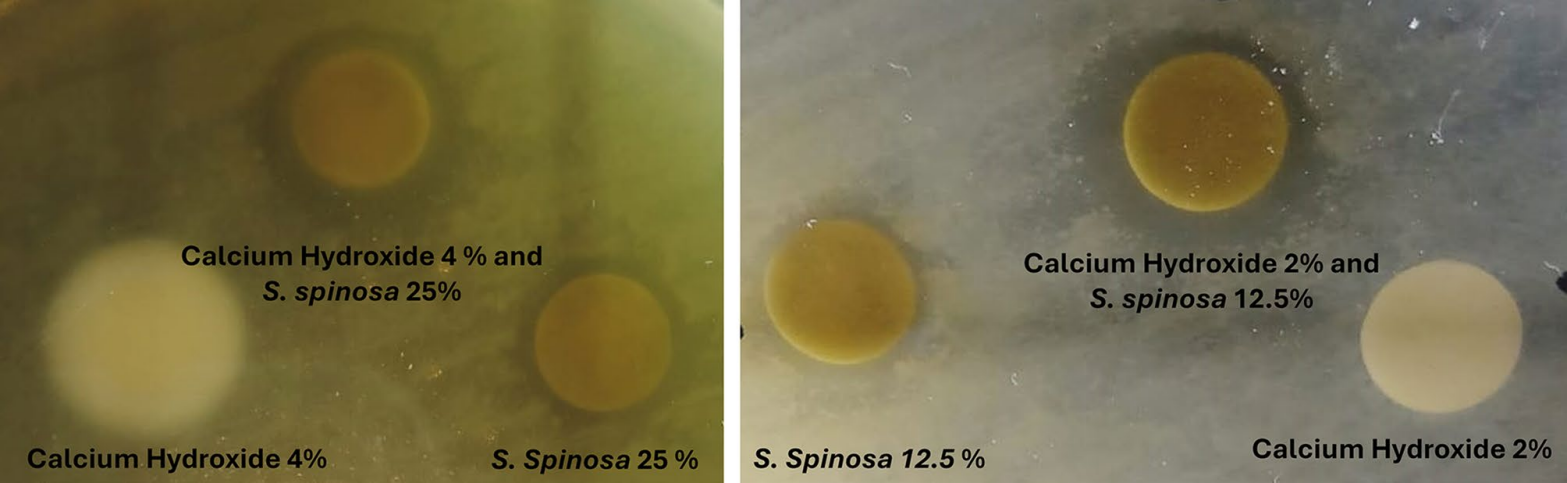
Disc Diffusion method showing the effect of *S. spinosa* extract and *S. spinosa*/Ca(OH)_2_ combination

Disc Diffusion test showed an increased zone of growth inhibition when combination of *S. spinosa* 25% extract and Calcium Hydroxide 4% was used [17 mm ± 2] as compared to *S. spinosa* 25% extract alone [12 mm ± 1]. Similarly, combination of *S. spinosa* 12.5% extract and Calcium Hydroxide 2% showed inhibition zones of [16 mm ± 3] as compared to *S. spinosa* 12.5% extract alone [10 mm ± 1]. Both Calcium Hydroxide 4% and Calcium Hydroxide 2% showed nearly no observable zone of growth inhibition (Fig. 1).

A two-way between-groups analysis of variance (ANOVA) was conducted to explore the impact of the type of intracanal medication used and time interval of medication application on counts of *Enterococcus faecalis* as measured by percent reduction in live colony counts. Studied teeth were divided into 3 groups according to the type of medication applied (Group 1: *Salvia spinosa* 25%; Group 2: Ca(OH)_2_ 4%; Group 3; Combination of *S. spinosa* 12.5% extract and Calcium Hydroxide 2%). A fourth group of ready-made Ca(OH)_2_ Paste was used as a control group.

**Fig. 2:**
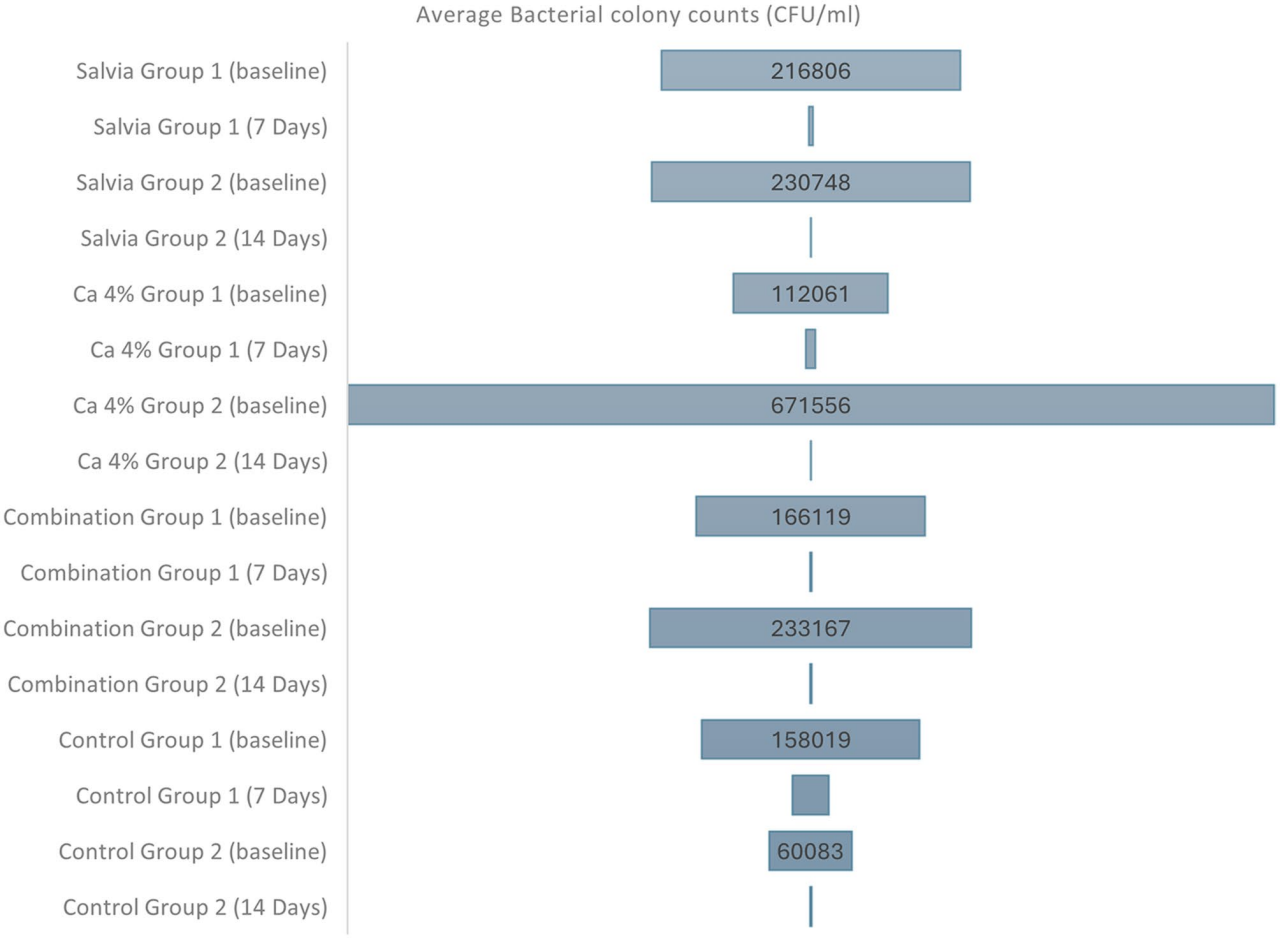
Comparative Effect of *Salvia spinosa* and Calcium Hydroxide studied experimental groups on reducing *E. faecalis* counts as compared to Ca(OH)_2_ Paste *Salvia* Group 1: *Salvia spinosa* extract 25% for 7 days, *Salvia* Group 2: *Salvia spinosa* extract 25% for 14 days, Ca 4% Group 1: Calcium Hydroxide 4% for 7 days, Ca 4% Group 2: Calcium Hydroxide 4% for 14 days, Combination Group 1: Combination of *Salvia spinosa* extract 12.5% and Calcium Hydroxide 2% for 7 days, Combination Group 2: Combination of *Salvia spinosa* extract 12.5% and Calcium Hydroxide 2% for 14 days, Control Group 1: Calcium Hydroxide commercial paste for 7 days, Control Group 2: Calcium Hydroxide commercial paste for 14 days

*E. faecalis* live colony counts for the studied groups at the two studied time intervals are shown in Table 1; Fig. 2.

**Table 1:** *Enterococcus faecalis* live colony counts (CFU/ml) for the studied groups

**Group 1 Medication**	**Salvia Group 1 baseline**	**Salvia Group 1** **7 Days (n = 6)**	**Salvia Group 2 baseline**	**Salvia Group 2** **14 Days (n = 6)**
Mean (CFU/ml)	216806	3089	230748	81
SD (CFU/ml)	310893	4098	188152	82
**Group 2 Medication**	**Ca (OH)**_**2**_ **4% Group 1 baseline**	**Ca (OH)**_**2**_ **4% Group 1 7 Days (n = 6)**	**Ca (OH)**_**2**_ **4% Group 2 baseline**	**Ca (OH)**_**2**_ **4% Group 2 ** **14 Days (n = 6)**
Mean (CFU/ml)	112061	6957	671556	26
SD (CFU/ml)	101828	3996	327465	20
**Group 3 Medication**	**Combination Group 1 baseline**	**Combination Group 1 ** **7 Days (n = 6)**	**Combination Group 2 baseline**	**Combination Group 2 ** **14 Days (n = 6)**
Mean (CFU/ml)	166119	1236	233167	1140
SD (CFU/ml)	149463	659	171686	1129
**Control Group**	**Ca (OH)**_**2**_ **Paste Group 1 baseline**	**Ca (OH)**_**2**_ **Paste Group 1 7 Days (n = 4)**	**Ca (OH)**_**2**_ **Paste Group 2 baseline**	**Ca (OH)**_**2**_ **Paste Group 2 14 Days (n = 4)**
Mean (CFU/ml)	158019	26481	60083	1308
SD (CFU/ml)	85929	28525	20272	1099

The interaction between the type of medication and time interval was not statistically significant, F (3, 44) = 2.585, *p* = 0.068. There was a statistically significant main effect for the type of medication used, F (3,44) = 3.774, *p* = 0.019 and also a statistically significant main effect for the time interval, F (1,44) = 19.584, *p* < 0.001 where more reduction was observed at the second time interval (14 days) as shown in Table 2. Levene’s Test of Equality of Error Variances was significant, indicating that variance across groups is not equal, so a more stringent significance level (0.01) was used to evaluate the results.

**Table 2:** Percent bacterial live colony count reduction among studied groups

Time Interval	Medication Group	Mean	Std. Deviation	*N*
7days	*Salvia* 25%	94.50	6.77	6
	Combination	96.94	5.46	6
	Ca (OH)_2_ 4%	90.25	7.77	6
	Ca (OH)_2_ Paste	80.32	15.40	4
	Total	91.43	10.08	22
14days	*Salvia* 25%	99.67	0.615	6
	Combination	98.98	1.66	6
	Ca (OH)_2_ 4%	99.99	0.0048	6
	Ca (OH)_2_ Paste	97.51	2.63	4
	Total	99.18	1.59	22
Total	*Salvia* 25%	97.09	5.32	12
	Combination	97.96	3.99	12
	Ca (OH)_2_ 4%	95.12	7.30	12
	Ca (OH)_2_ Paste	88.91	13.75	8
	Total	95.30	8.13	44

Post-hoc comparisons using the Dunnett test indicated that percent reduction in the combination group (M = 97.96, SD = 3.99) was statistically significantly different (*p* = 0.009) from the control group (M = 88.92, SD = 13.75). Percent reduction also showed statistically significant difference (*p* = 0.019) for *Salvia* treatment (M = 97.09, SD = 5.32) as compared to control group but did not show significant difference (*p* = 0.089) for Ca(OH)_2_ 4% treatment (M = 95.12, SD = 7.3) as compared to control group.

*Salvia spinosa* 25% extract showed significantly better antibacterial effect than the standard calcium hydroxide paste. The combination of calcium hydroxide 2% and *S. spinosa* 12.5% extract also showed better antibacterial effect than calcium hydroxide 4% alone and also better than standard calcium hydroxide paste. Combination of calcium hydroxide 2% and *S. spinosa* 12.5% extract showed higher mean percentage reduction in live bacterial counts at 7 days compared to *Salvia* alone, however the overall difference between *Salvia* and combination (Tukey Post hoc HSD comparison) was not statistically significant (*p* = 0.986).

Analysis of in-silico interactions showed that 6 *S. spinosa* compounds among the studied 19 compounds exhibit the highest affinities to *E. faecalis* proteins studied. Those included Flavone, Flavonone, Caryophyllene oxide, Spathulenol, β-patchoulene, and alpha-Gurjunene. Binding energies for these compounds and types of interactions with target proteins are shown in Tables 3, 4, 5 and 6. Diagrams showing interaction between *E. faecalis* protein macromolecules with best binding compounds are shown in Supplementary Figs. 1–3. Both flavonone and flavone showed the highest binding affinities to Enterococcal surface protein (6ORI), Adhesin domain of PrgB (6EVU), collagen adhesion protein (2Z1P), Aggregation substance PrgB/Glucan-binding protein (M1U904) and also to Peptide pheromone-binding protein TraC (A0A6I4Y5I4). Similarly, Caryophyllene oxide and Spathulenol showed the highest binding affinities to both Enterococcal surface protein (6ORI) and to collagen adhesion protein (2Z1P).

**Table 3:** Interaction and binding affinity of flavone and flavonone with *E. faecalis* surface proteins

Ligand (S. spinosa Active component)	Macromolecule (E. faecalis protein)	Binding Energy (Kcal/mol)	Reference RMSD	Inhibition constant (Ki)	No of Hydrophobic interactions and Amino acids involved in interaction	No of H bonds (drug-protein) and Amino acids involved in interaction
Flavone	Enterococcal surface protein (6ORI)	**-8.3**	5.496 A	826.11 nM	5 [86 A LYS, 238 A LEU (3), 442 A]	1 [236 A TYR]
	Adhesin domain of PrgB (6EVU)	**-8.2**	46.939 A	983.34 nM	4 [256 A ASN, 475 A LYS (2), 557 A LYS]	1 [476 A LYS]
	collagen adhesion protein (2Z1P)	**-7.69**	104.071 A	2.30 μm	8 [185 A LEU (2), 190 A ASN, 282 A ILE (2), 287 A GLN, 319 A VAL, 321 A VAL]	----------------------
	Enterococcal surface protein *esp* (F6MHT4)	-6.54	22.375 A	16.07 μm	3 [13 A LYS, 14 A ALA, 18 A ILE]	2 [21 A ASN, 22 A ALA]
	Peptide pheromone-binding protein TraC (A0A6I4Y5I4)	**-7.7**	10.474 A	2.27 μm	7 [141 A GLU (2), 142 A LEU, 145 A ALA, 450 A TRP, 458 A ILE, 462 A ALA]	------------------------
	*Enterococcus faecalis* protein Asa1 (M1TU09)	-6.25	10.930 A	26.09 μm	6 [49 A LEU, 72 A TYR (2), 137 A VAL, 275 A TRP (2)]	2 [49 A LEU, 72 A TYR]
	Endocarditis and biofilm-associated pilus major subunit EbpC (A0A7H0FRK3)	-7.24	64.571 A	4.95 μm	2 [161 A TYR, 173 A TYR]	1 [157 A ALA]
	collagen adhesin (Ace) (E1ART9)	-6.72	6.238 A	11.85 μm	7 [10 A TYR, 14 A MET, 144 A ASP, 145 A LEU (2), 276 A VAL 279 A VAL]	1 [153 A ARG]
	Aggregation substance PrgB/Glucan-binding protein (M1U904)	**-7.65**	22.580 A	2.47 μm	5 [24 A ASN, 117 A LEU, 310 A PRO, 312 A PHE, 313 A ASN]	1 [313 A ASN]
Flavonone	Enterococcal surface protein (6ORI)	**-8.94**	6.016 A	280.28 nM	7 [85 A PRO, 86 A LYS, 87 A ALA, 238 A LEU (3), 442 A THR]	2 [214 A TYR, 236 A TYR]
	Adhesin domain of PrgB (6EVU)	**-8.23**	48.023 A	928.02 nM	5 [256 A ASN, 468 A TYR, 475 A LYS (2), 557 A LYS]	1 [476 A LYS]
	collagen adhesion protein (2Z1P)	**-7.96**	104.230 A	1.47 μm	8 [185 A LEU (2), 190 A ASN, 282 A ILE (2), 287 A GLN, 319 A VAL, 321 A VAL]	1 [190 A ASN]
	Enterococcal surface protein *esp* (F6MHT4)	-7	15.702 A	7.34 μm	4 [23 A PRO, 79 A GLU, 93 A LYS, 134 A THR]	1 [77 A LYS]
	Peptide pheromone-binding protein TraC (A0A6I4Y5I4)	**-7.64**	11.516 A	2.52 μm	6 [141 A GLU (2), 142 A LEU, 450 A TRP, 458 A ILE, 462 A ALA]	1 [454 A TYR]
	*Enterococcus faecalis* protein Asa1 (M1TU09)	-6.28	11.904 A	25.12 μm	6 [183 A ASN, 190 A THR, 192 A GLU, 232 A ILE, 269 A TYR, 271 A GLY]	------------------------
	Endocarditis and biofilm-associated pilus major subunit EbpC (A0A7H0FRK3)	-7.29	64.454 A	4.51 μm	3 [160 A VAL, 161 A TYR, 173 A TYR]	3 [157 A ALA, 161 A TYR, 173 A TYR]
	collagen adhesin (Ace) (E1ART9)	-6.6	6.398 A	14.55 μm	5 [13 A GLU, 14 A MET, 144 A ASP, 276 A VAL, 277 A LYS]	1 [14 A MET]
	Aggregation substance PrgB/Glucan-binding protein (M1U904)	**-8.2**	22.623 A	968.87 nM	6 [24 A ASN, 117 A LEU, 310 A PRO, 312 A PHE (2), 314 A TYR]	1 [313 A ASN]

**Table 4:** Interaction and binding affinity of spathulenol and β-patchoulene with *E. faecalis* surface proteins

Ligand (S. spinosa Active component)	Macromolecule (E. faecalis protein)	Binding Energy (Kcal/mol)	Reference RMSD	Inhibition constant (Ki)	No of Hydrophobic interactions and Amino acids involved in interaction	No of H bonds (drug-protein) and Amino acids involved in interaction
Spathulenol	Enterococcal surface protein (6ORI)	**-8.06**	15.075 A	1.24 μm	5 [66 A PHE, 123 A GLU, 213 A MET, 446 A TYR (2)]	6 [64 A ASN, 65 A THR (3), 66 A PHE (2)]
	Adhesin domain of PrgB (6EVU)	-7.53	47.052 A	3.04 μm	9 [466 A PHE (2), 468 A TYR, 475 A LYS, 482 A PHE (2), 557 A LYS (3)]	1 [261 A GLU]
	collagen adhesion protein (2Z1P)	**-7.73**	102.272 A	2.14 μm	7 [86 A MET, 185 A LEU (2), 282 A ILE , 87 A GLN, 321 A VAL]	4 [187 A GLY, 190 A ASN (2), 191 A GLN]
	Enterococcal surface protein *esp* (F6MHT4)	-6.84	15.444 A	9.67 μm	7 [21 A ASN, 59 A ASP, 79 A GLU, 93 A LYS (2), 95 A PHE, 134 A THR]	1 [134 A THR]
	Peptide pheromone-binding protein TraC (A0A6I4Y5I4)	-6.56	3.662 A	15.51 μm	4 [60 A VAL, 62 A GLN, 450 A TRP, 451 A GLN]	1 [60 A VAL]
	*Enterococcus faecalis* protein Asa1 (M1TU09)	-6.33	12.390 A	22.76 μm	7 [49 A LEU (2), 50 A ILE (2), 72 A TYR, 137 A VAL (2)]	2 [49 A LEU, 72 A TYR]
	Endocarditis and biofilm-associated pilus major subunit EbpC (A0A7H0FRK3)	-6.81	61.617 A	10.26 μm	4 [58 A ASN, 156 A VAL, 160 A VAL, 183 A ILE]	2 [57 A GLN (2)]
	collagen adhesin (Ace) (E1ART9)	-5.93	5.566 A	44.70 μm	3 [10 A TYR, 144 A ASP, 276 A VAL]	2 [10 A TYR, 14 A MET]
	Aggregation substance PrgB/Glucan-binding protein (M1U904)	-7	8.199 A	7.36 μm	4 [262 A ALA, 296 A TRP, 298 A ALA, 302 A ASN]	2 [194 A SER, 304 A ASN]
β-patchoulene	Enterococcal surface protein (6ORI)	-7.91	13.996 A	1.60 μm	5 [65 A THR, 123 A GLU (2), 444 A TYR, 446 A TYR]	---------------------------
	Adhesin domain of PrgB (6EVU)	-6.91	47.964 A	8.61 μm	7 [252 A VAL, 256 A ASN, 468 A TYR (3), 475 A LYS, 476 A LYS]	---------------------------
	collagen adhesion protein (2Z1P)	-6.96	103.409 A	7.91 μm	6 [185 A LEU 282 A ILE (2), 287 A GLN, 321 A VAL (2)]	---------------------------
	Enterococcal surface protein *esp* (F6MHT4)	-6.55	14.745 A	15.89 μm	7 [59 A ASP, 77 A LYS, 79 A GLU, 95 A PHE (3), 134 A THR]	
	Peptide pheromone-binding protein TraC (A0A6I4Y5I4)	-6.01	13.467 A	39.36 μm	6 [279 A ALA, 337 A LEU, 403 A PHE, 512 A PHE, 514 A VAL, 516 A THR]	
	*Enterococcus faecalis* protein Asa1 (M1TU09)	-6.03	12.272 A	37.74 μm	10 [48 A LEU (2) 49 A LEU (2), 50 A ILE (3), 72 A TYR (2), 137 A VAL]	
	Endocarditis and biofilm-associated pilus major subunit EbpC (A0A7H0FRK3)	-6.27	62.350 A	25.28 μm	4 [58 A ASN, 160 A VAL, 173 A TYR, 183 A ILE]	
	collagen adhesin (Ace) (E1ART9)	-5.74	30.777 A	62.39 μm	6 [21 A PHE, 24 A LYS, 29 A ILE, 33 A ASP, 35 A ILE (2)]	
	Aggregation substance PrgB/Glucan-binding protein (M1U904)	-6.43	7.166 A	19.23 μm	6 [149 A ILE, 191 A ALA, 257 A VAL (2), 262 A ALA, 298 A ALA]	

**Table 5:** Interaction and binding affinity of caryophyllene oxide and alpha-Gurjunene with *E. faecalis* surface proteins

Ligand (S. spinosa Active component)	Macromolecule (E. faecalis protein)	Binding Energy (Kcal/mol)	Reference RMSD	Inhibition constant (Ki)	No of Hydrophobic interactions and Amino acids involved in interaction	No of H bonds (drug-protein) and Amino acids involved in interaction
Caryophyllene oxide	Enterococcal surface protein (6ORI)	**-8.48**	14.850 A	611.64 nM	8 [64 A ASN, 65 A THR, 66 A PHE, 122 A ASP, 123 A GLU, 444 A TYR, 446 A TYR (2)]	2 [123 A GLU, 159 A ASN]
	Adhesin domain of PrgB (6EVU)	-7.29	47.133 A	4.54 μm	8 [466 A PHE, 467 A LYS, 468 A TYR (2), 475 A LYS, 482 A PHE 557 A LYS (2)]	1 [476 A LYS]
	collagen adhesion protein (2Z1P)	**-7.94**	101.089 A	1.51 μm	4 [185 A LEU, 319 A VAL, 321 A VAL (2)]	-------------------
	Enterococcal surface protein *esp* (F6MHT4)	-7.42	15.689 A	3.64 μm	5 [21 A ASN, 59 A ASP, 77 A LYS, 95 A PHE (2)]	1 [95 A PHE]
	Peptide pheromone-binding protein TraC (A0A6I4Y5I4)	-6.62	13.299 A	13.95 μm	5 [279 A ALA, 337 A LEU, 512 A PHE, 514 A VAL, 516 A THR]	1 [517 A ALA]
	*Enterococcus faecalis* protein Asa1 (M1TU09)	-6.74	11.892 A	11.46 μm	10 [49 A LEU, 50 A ILE, 72 A TYR (2), 137 A VAL (2) 275 A TRP (4)]	1 [72 A TYR]
	Endocarditis and biofilm-associated pilus major subunit EbpC (A0A7H0FRK3)	-6.61	61.484 A	14.19 μm	6 [156 A VAL (2), 160 A VAL, 173 A TYR, 181 A VAL, 183 A ILE]	1 [173 A TYR]
	collagen adhesin (Ace) (E1ART9)	-6.21	30.576 A	28.19 μm	5 [27 A GLN, 29 A ILE, 33 A ASP, 35 A ILE, 75 A PHE]	1 [23 A ASP]
	Aggregation substance PrgB/Glucan-binding protein (M1U904)	-7.18	7.023 A	5.43 μm	4 [149 A ILE, 257 A VAL, 298 A ALA, 302 A ASN]	2 [300 A SER, 302 A ASN]
alpha-Gurjunene	Enterococcal surface protein (6ORI)	-7.67	5.527 A	2.40 μm	4 [238 A LEU (2), 440 A TYR, 442 A THR]	-------------------------
	Adhesin domain of PrgB (6EVU)	-7.09	46.179 A	6.30 μm	9 [252 A VAL, 253 A ALA, 256 A ASN, 466 A PHE, 468 A TYR (2), 475 A LYS (2), 476 A LYS]	------------------------
	collagen adhesion protein (2Z1P)	-7.25	102.522 A	4.82 μm	10 [185 A LEU (3), 190 A ASN, 282 A ILE (2), 287 A GLN, 319 A VAL, 321 A VAL (2)]	------------------------
	Enterococcal surface protein *esp* (F6MHT4)	-6.64	14.947 A	13.68 μm	7 [21 A ASN, 59 A ASP, 77 A LYS, 93 A LYS, 95 A PHE (2), 134 A THR]	--------------------------
	Peptide pheromone-binding protein TraC (A0A6I4Y5I4)	-6.64	2.304 A	13.50 μm	5 [60 A VAL, 62 A GLN, 65 A ILE, 450 A TRP, 451 A GLN]	-------------------------
	*Enterococcus faecalis* protein Asa1 (M1TU09)	-6.04	17.470 A	37.22 μm	4 [242 A GLU, 243 A TYR, 283 A LYS, 285 A LYS]	--------------------------
	Endocarditis and biofilm-associated pilus major subunit EbpC (A0A7H0FRK3)	-6.37	61.148 A	21.45 μm	6 [58 A ASN, 156 A VAL, 160 A VAL, 173 A TYR, 181 A VAL, 183 A ILE]	------------------------
	collagen adhesin (Ace) (E1ART9)	-5.62	30.623 A	75.86 μm	4 [27 A GLN, 29 A ILE, 33 A ASP, 75 A PHE]	-------------------------
	Aggregation substance PrgB/Glucan-binding protein (M1U904)	-6.73	7.292 A	11.72 μm	5 [257 A VAL, 262 A ALA, 296 A TRP (2), 298 A ALA]	-------------------------

**Table 6:** Aromatic interactions (π-π aromatic stacking) identified at the tested ligand- *E. faecalis* surface proteins interface

Ligand	Protein	AA Residue	Distance	Iteraction Type	Ligand Group	Ligand Atoms
FLavone	2z1p	94 A ARG	4.57	π-Cation Interactions	Aromatic	2677, 2679, 2680, 2681, 2682, 2683
FLavone	6evu	468 A TYR	4.34	π-Stacking (Stacking type P)	Angle 11.84	2994, 2995, 2996, 2997, 2998, 2999
FLavone	6ori	214 A TYR	5.3	π-Stacking (Stacking type T)	Angle 71.23	3947, 3949, 3952, 3953, 3954, 3955
FLavone	6ori	214 A TYR	5.38	π-Stacking (Stacking type T)	Angle 71.16	3945, 3947, 3948, 3949, 3950, 3951
FLavone	F6MHT4	13 A LYS	3.58	π-Cation Interactions	Aromatic	2797, 2799, 2802, 2803, 2804, 2805
FLavone	M1TU09	275 A TRP	5.14	π-Stacking (Stacking type T)	Angle 88.33	2790, 2792, 2795, 2796, 2797, 2798
FLavone	M1TU09	275 A TRP	5.09	π-Stacking (Stacking type T)	Angle 88.12	2790, 2792, 2795, 2796, 2797, 2798
Flavonone	6ori	214 A TYR	5.46	π-Stacking (Stacking type T)	Angle 69.13	3950, 3951, 3952, 3953, 3954, 3955
Flavonone	6evu	468 A TYR	4.36	π-Stacking (Stacking type P)	Angle 17.83	2994, 2995, 2996, 2997, 2998, 2999
Flavonone	F6MHT4	95 A PHE	4.14	π-Stacking (Stacking type P)	Angle 17.44	2806, 2807, 2808, 2809, 2810, 2811

## Discussion

Calcium hydroxide (Ca(OH)_2_) is the most conventional temporary root canal medicament used to prevent bacterial re-growth in root canals between treatment sessions. *Enterococcus faecalis* is a highly resistant bacteria capable of enduring harsh conditions and elevated pH levels, and it has been frequently implicated as a fundamental cause of endodontic treatment failures. Previous studies have shown that Ca(OH)_2_ has a limited effect against *E. faecalis* in root canal infections [36].

The combination treatment minimizes the concentrations and clinical doses of drugs, hence decreasing potential toxicities while facilitating a potentially synergistic or improved therapeutic impact. Chlorhexidine has been used in combination with Ca(OH)_2_ to kill root canal *E. faecalis*. While Chlorhexidine gel showed better effect than Ca(OH)_2_ alone, Chlorhexidine Ca(OH)_2_ combination did not enhance the antibacterial effect [37]. Evidence also showed that mixing Ca(OH)_2_ with Chlorhexidine does not significantly increase the antimicrobial activity of Ca(OH)_2_ against *E. faecalis* [38]. In other more recent studies, chlorhexidine gel, chitosan gel, and silver nanoparticle gel combinations with Ca(OH)_2_ showed better antibacterial activity against *E. faecalis* with bioactive glass S53P4 combination showing the maximum effect [39].

Several natural compounds have been tested for their antibacterial effect on *E. faecalis* compared to standard Ca(OH)_2_. Combinations of cinnamon and ginger extracts have shown antibacterial effect against *E. faecalis* but the effect was not statistically significantly different from that of Ca(OH)_2_ [40]. Other studies have shown that the antimicrobial effect of Calcium Hydoxide against *E. faecalis* was not enhanced by its combination with *Nigella sativa* extract [41]. In other studies, researchers have shown that both honey and turmeric “*Curcuma longa*” exhibited better antimicrobial effect than Ca(OH)_2_ against *E. faecalis* [42]. Another study has demonstrated the promising effect of the Chinese herbal extract Ginsenoside in combination with Ca(OH)_2_ against *E. faecalis* [43]. In the another study, *Spilanthesacmella* and *cinnamon oil* showed promising antibacterial effect when compared to Ca(OH)_2_ against *E. faecalis* [44] Another study has demonstrated the superior effect of *Aloe vera* when compared to Calcium Hydroxide against *E. faecalis* [45].

In this study we have tested the effect of *S. spinosa* extract on killing *E. faecalis* causing endodontic infections in an ex-vivo model and we have also tested the antibacterial effect of combining *S. spinosa* extract with Ca(OH)_2_. Both *Salvia* extract and *S. spinosa*- Ca(OH)_2_ combination exhibited similar superior antibacterial effects compared to standard Calcium Hydroxide paste with the advantage of achieving similar antibacterial effects against *E. faecalis* at lower concentrations of the active substances when the combination was used. Although the enhanced combinatorial effect was obvious with disc diffusion testing, combinatorial effect was not significantly different from effect of *S. spinosa* tested alone in the ex-vivo model which indicates a possible chemical interaction of *S. spinosa* with calcium, however, confirming the presence of such interaction and its exact mechanism requires further investigation.

Supporting these findings, a recent study has demonstrated a synergistic antimicrobial effect of a unique combination of hydroalcoholic extracts originating from *Glycyrrhiza glabra* and *Salvia officinalis* against *Enterococcus faecium and Enterococcus faecalis* biofilms with an effect comparable to that of a 0.12% chlorhexidine solution [46]. Additionally, another study has demonstrated the promising antibacterial effect of the essential oil of *Salvia officinalis* growing in Morocco against *Enterococcus faecalis* [47]. Earlier studies have also shown the potential of *Salvia officinalis* plant extract as an endodontic irrigant with reported lower bactericidal action than chlorhexidine with recommending further testing as an intracanal medicament [48]. Other studies have also demonstrated a good antimicrobial effect of *Salvia officinalis* L. against Gram positive organisms including *E. faecalis* [49]. However, in another study, *Salvia officinalis* demonstrated a minimal antimicrobial activity against *E. faecalis* using Disc diffusion method being the lowest among the other Gram-positive organisms studied [50].

Equipped with several virulence factors such as pheromones, aggregation compounds, cytolysin, lytic enzymes, adhesion proteins, and lipoteichoic acid, *E. faecalis* can persist in root canal systems, leading to recurrent infections. Antibacterial agents that target critical adhesion proteins would be highly beneficial in combating such problematic resistant species. Literature shows that there is no satisfactory method for eliminating *E. faecalis* root canal infections once established and for that reason inhibiting initial attachment of the bacterium is an alternative, better approach than eliminating an already established infection as previously suggested [51]. Aggregation substances serve as adhesins that enable *E. faecalis* to adhere to different matrix proteins playing an important role in host infection. Asa1 is among the aggregation substances having a role in attachment to extracellular matrix components including fibrin, fibronectin, thrombospondin, vitronectin and collagen type 1 and is also important in invasion [52]. Evidence shows that aggregation substances facilitate adherence to endothelium and non-intact epithelium, in which extracellular matrix proteins are exposed, thereby promoting colonization and infection [53]. Esp is thought to have a role in protecting bacterial cells against immune responses and is more frequently encountered in clinical isolates causing nosocomial infections [54]. Studies have also demonstrated that Esp plays a significant role in biofilm formation [55]. Ace termed as “adhesin of collagen from *E. faecalis*” was one of the first surface adhesion proteins described in *Enterococci*. It facilitates binding to dentin, laminin, collagen type I, and collagen type IV [56].

In this study, we have tested the in-silico interaction of *S. spinosa* active constituent with important adhesion and surface proteins of *E. faecalis* with the aim of finding probable drug targets and promising active compounds to act as further candidates for new tailored intracanal medicaments.

Overall, flavone and flavonone showed the best binding to *E. faecalis* surface proteins studied followed by Spathulenol and Caryophyllene oxide. The best binding *E. faecalis* proteins to all ligands included both Enterococcal surface protein (6ORI), Adhesin domain of PrgB (6EVU), and the collagen adhesion protein (2Z1P). Peptide pheromone-binding protein TraC (A0A6I4Y5I4), Aggregation substance PrgB/Glucan-binding protein (M1U904), and Enterococcal surface protein *esp* (F6MHT4) also showed high binding affinities to the 6 best binding ligands concluding that these proteins may be promising drug or vaccine targets for preventing the establishment and progression of *E. faecalis* in root canals.

PrgB is a main adhesin in *E. faecalis* and is widely distributed over all the surface of the cell wall increasing biofilm formation and cellular aggregation. It also increases the efficiency of plasmid transfer and is considered a potent virulence factor. Being identified in many other conjugative plasmids, it is suggested that PrgB-like proteins may have important roles in various bacterial species [57].

Interestingly, flavone and flavonones have shown π–π stacking and cation-π interactions (Table 5) with different Enterococcal proteins. It has been a relatively broadly agreed that π–π stacking interactions could efficiently improve the conformational stability of various chemical systems [58]. Moreover, π–π and cation-π interactions were also proposed to have a very essential job in rational drug design [58, 59].

Although the study has investigated in detail the effect of *S. spinosa* on *E. faecalis* using in-vitro, ex-vivo, and in-silico approach, some limitations should be acknowledged. The study used a simplified monomicrobial teeth model which requires future *in-vivo* validation. Although *E. faecalis* is the most resistant and troublesome endodontic microbe encountered, the clinical scenario of root canal infections may involve multispecies etiology. In that respect, future studies incorporating more complex multispecies infection models and in-vivo studies will be useful to evaluate the overall efficacy of *S. spinosa* in clinical scenarios.

## Conclusion

Exhibiting a good in-vitro, and ex-vivo antibacterial effect against *E. faecalis*, *S. spinosa* offers a natural potential candidate for a new intracanal medication effective against one of the most challenging endodontic pathogens. In-silico findings also point to the promising potential of its most active constituent in targeting the most important surface adhesins and pathogenicity factors which allows further synthetic tailoring into promising drug candidates that help in preventing initial establishment and further progression of *E. faecalis* colonization in root canal systems. Further cytotoxicity studies are also recommended for further tailoring of its therapeutic potential.

## Electronic supplementary material

Below is the link to the electronic supplementary material.

Supplementary Material 1

## Data Availability

All data generated and analyzed in this work are presented in manuscript and its associated supplementary information.

## Abbreviations

Ca(OH)_2_: Calcium Hydroxide
CFUs: Colony forming units
ANOVA: Analysis of variance
RMSD: Root Mean Square Deviation
