# Correction: Antimicrobial activity of *Salvia spinosa* against *Enterococcus faecalis* causing endodontic infections: an in-vitro, ex-vivo, and in-silico study

**DOI:** 10.1186/s12906-025-05069-5

**Published:** 2025-09-12

**Authors:** Wedad M. Nageeb, Sherouk Hussein Adam, Nihal Ali, Marwa Sharaan

**Affiliations:** 1 Department of Medical Microbiology and Immunology, Faculty of Medicine, Suez Canal University, Ismailia, Egypt; 2 Department of Endodontics, Faculty of Dentistry, Suez Canal University, Ismailia, Egypt; 3 Directorate of Health, Suez, Egypt

**Correction: BMC Complement Med Ther 25, 254 (2025)**

**https://doi.org/10.1186/s12906-025-04983-y**

Following publication of the original article [1], the authors reported an error in Table 1. The columns titles are incorrectly formatted. The correct table is given below.

**Table 1:** *Enterococcus faecalis* live colony counts (CFU/ml) for the studied groups

**Group 1 Medication**	**Salvia Group 1 baseline**	**Salvia Group 1** **7 Days (** ***n*** **= 6)**	**Salvia Group 2 baseline**	**Salvia Group 2** **14 Days (** ***n*** **= 6)**
Mean (CFU/ml)	216,806	3089	230,748	81
SD (CFU/ml)	310,893	4098	188,152	82
**Group 2 Medication**	**Ca (OH)**_**2**_ **4% Group 1 baseline**	**Ca (OH)**_**2**_ **4% Group 1 7 Days (** ***n*** ** = 6)**	**Ca (OH)**_**2**_ **4% Group 2 baseline**	**Ca (OH)**_**2**_ **4% Group 2 ** **14 Days (** ***n*** **= 6)**
Mean (CFU/ml)	112,061	6957	671,556	26
SD (CFU/ml)	101,828	3996	327,465	20
**Group 3 Medication**	**Combination Group 1 baseline**	**Combination Group 1 ** **7 Days (** ***n*** **= 6)**	**Combination Group 2 baseline**	**Combination Group 2 ** **14 Days (** ***n*** **= 6)**
Mean (CFU/ml)	166,119	1236	233,167	1140
SD (CFU/ml)	149,463	659	171,686	1129
**Control Group**	**Ca (OH)**_**2**_ **Paste Group 1 baseline**	**Ca (OH)**_**2**_ **Paste Group 1 7 Days (** ***n*** ** = 4)**	**Ca (OH)**_**2**_ **Paste Group 2 baseline**	**Ca (OH)**_**2**_ **Paste Group 2 14 Days (** ***n*** ** = 4)**
Mean (CFU/ml)	158,019	26,481	60,083	1308
SD (CFU/ml)	85,929	28,525	20,272	1099
