# Supplementary material for: Antimicrobial activity of Salvia spinosa against Enterococcus faecalis causing endodontic infections: an in-vitro, ex-vivo, and in-silico study
Source: BMC Complement Med Ther. 2025 Jul 9;25:254. doi: 10.1186/s12906-025-04983-y (PMC12243204; doi:10.1186/s12906-025-04983-y)
Supplement: Supplementary file 1 — Supplementary Material 1 [file 12906_2025_4983_MOESM1_ESM.pdf]

Title

Antimicrobial Activity of *Salvia spinosa* Against *Enterococcus faecalis* causing endodontic infections: An In-Vitro, Ex-Vivo, and In-Silico Study

Authors

Wedad M. Nageeb[1]*, Sherouk Hussein Adam[2], Nihal Ali[3], Marwa Sharaan[4]

Affliations

[1]Department of Medical Microbiology and Immunology, Faculty of Medicine, Suez Canal University, Ismailia, Egypt, wedad_saleh@med.suez.edu.eg

[2]Department of Endodontics, Faculty of Dentistry, Suez Canal University, Ismailia, Egypt, Shrouk_hussein@dent.suez.edu.eg

[3]Directorate of Health, Suez, Egypt,

Nihalali34549@gmail.com

[4]Department of Endodontics, Faculty of Dentistry, Suez Canal University, Ismailia, Egypt

Marwaelsayedsharaan@gmail.com

***Correspondence: Wedad M. Nageeb,** Department of Medical Microbiology and Immunology, Faculty of Medicine, Suez Canal University, Ismailia, Egypt

E-mail: wedad_saleh@med.suez.edu.eg

Supplementary Figure 1: Interactions of *S. spinosa* active compounds showing highest binding affinities with Enterococcal surface protein (6ORI) of *Enterococcus faecalis*

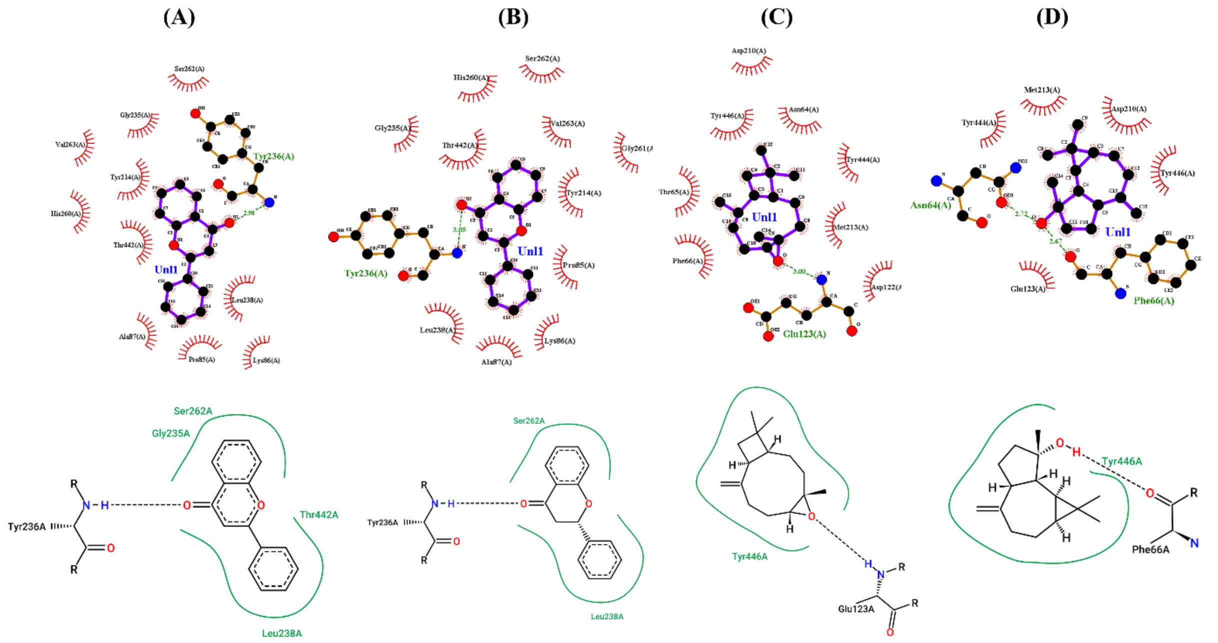

(A) Flavone: Binding Energy = - 8.3 kcal/mol
(B) Flavonone: Binding Energy = - 8.94 kcal/mol
(C) Caryophyllene oxide: Binding Energy = - 8.48 kcal/mol
(D) Spathulenol: Binding Energy = - 8.06 kcal/mol

Supplementary Figure 2: Interactions of *S. spinosa* active compounds showing highest binding affinities with Adhesin domain of PrgB (6EVU) of *Enterococcus faecalis*

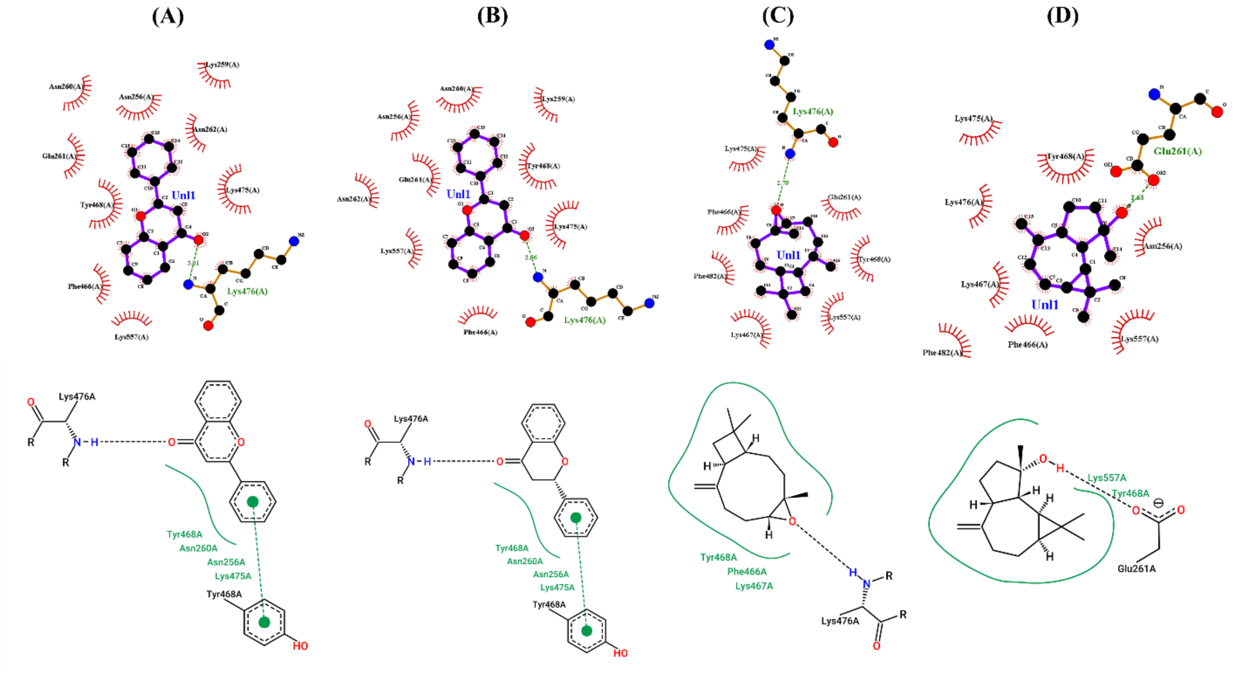

(A) Flavone: Binding Energy = - 8. kcal/mol
(B) Flavonone: Binding Energy = - 8.23 kcal/mol
(C) Caryophyllene oxide: Binding Energy = - 7.29 kcal/mol
(D) Spathulenol: Binding Energy = - 7.53 kcal/mol

Supplementary Figure 3: Interactions of *S. spinosa* active compounds showing highest binding affinities with Aggregation substance PrgB/Glucan-binding protein (M1U904) of *Enterococcus faecalis*

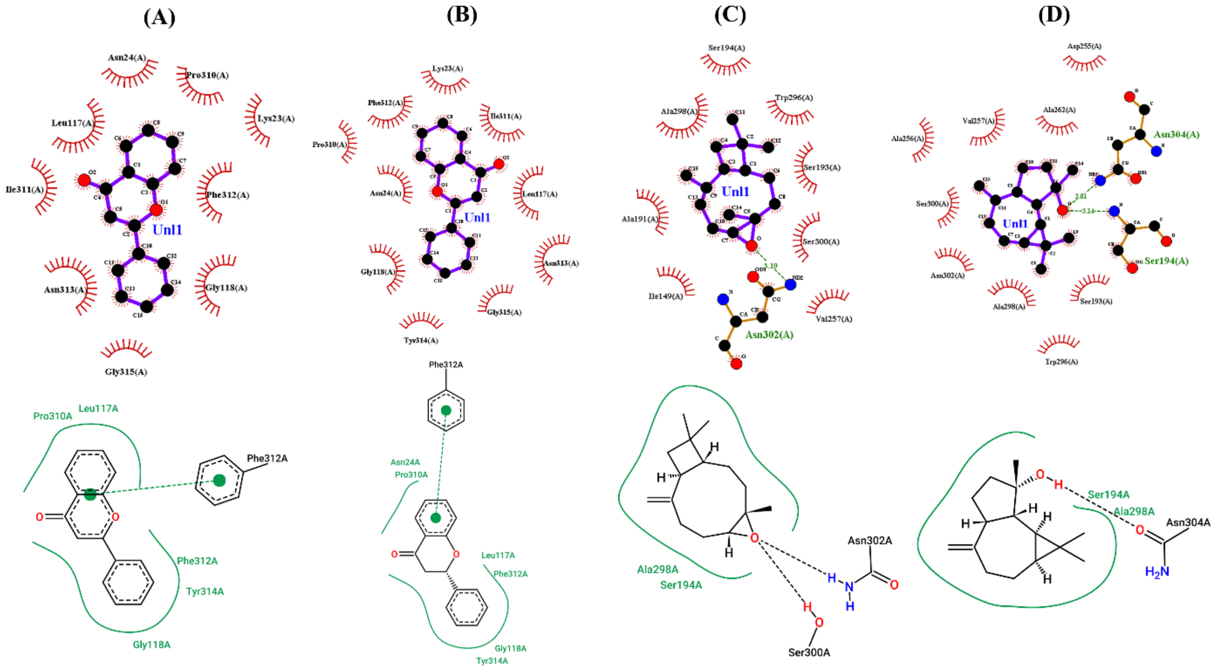

(A) Flavone: Binding Energy = - 7.65. kcal/mol
(B) Flavonone: Binding Energy = - 8.2 kcal/mol
(C) Caryophyllene oxide: Binding Energy = - 7.18 kcal/mol
(D) Spathulenol: Binding Energy = - 7 kcal/mol
